# Factors Influencing Length of Stay and Discharge Destination of Patients with Hip Fracture Rehabilitating in a Private Care Setting

**DOI:** 10.3390/geriatrics7020044

**Published:** 2022-03-31

**Authors:** Zoe Thornburgh, Dinesh Samuel

**Affiliations:** Faculty of Environmental and Life Sciences, University of Southampton, Highfield, Southampton SO17 1BJ, UK

**Keywords:** hip fracture, length of stay, rehabilitation, discharge, delirium

## Abstract

**Background:** Rehabilitation after a hip fracture has long-term importance, prompting some patients to utilise private services. Insufficient data regarding private rehabilitation in the UK can cause ambiguity and potential problems for all involved. **Aim:** The present study, involving patients with hip fractures rehabilitating in a private UK care setting, examined relationships between length of stay (LoS), discharge destination (DD) and 12 predictor variables. **Methods:** The variables included the retrospective measurement of the Functional Independence Measure. The variables were informed by a literature review and patient and public involvement. Retrospective data from the records of patients with hip fractures were utilised. Data were analysed using Spearman’s rho, Mann–Whitney U, Kruskal–Wallis H and chi-squared tests as appropriate. Odds ratios, distribution quartiles and survivor analysis were also utilised. **Results:** The median length of stay (LoS) was 20.5 days: 82% returned home, 6.5% died and 11.5% remained as long-term residents. Significant relationships existed between LoS and age (*p* = 0.004), comorbidities (*p* = 0.001) and FIM_admission_ (*p* = 0.001). DD was associated with age (*p* = 0.007), delirium (*p* = 0.018), comorbidities (*p* = 0.001) and both FIM_pre-fracture_ and FIM_admission_ (*p* = 0.000). **Conclusions:** Factors associated with length of stay were identified, but further research incorporating multiple sites is required for greater predictor precision. Discharge destination was evident by 90 days, facilitating long-term planning.

## 1. Introduction

Hip fracture is the most common serious injury in older people [1], creating long-lasting issues for patients and health services alike. Full recovery is uncommon, and physical function often significantly and permanently declines [2,3]. In 2017, only 67.5% of patients with a hip fracture returned to their original residence four months post-injury, and 9% were still immobile [4]. Recovery and independence improve with rehabilitation [5], which should start on the first day post-surgery [6]. Without this, patients are slower to discharge [7], less likely to walk independently or live at home after one year [8] and have a greater risk of psychological issues [9] and mortality [10,11,12].

Within the UK, approximately 95% of hip fractures present to the National Health Service (NHS) [13], with most remaining there for the full treatment process. Optimally, rehabilitation should continue beyond the acute inpatient stage [14]. A limited number of inpatient beds and long community delays [4] result in rehabilitation being based on availability rather than patient requirements [15]. Recognising the importance of prompt, continuous and accessible treatment [16,17], some patients take charge and choose private rehabilitation services [17].

There is a wealth of hip fracture rehabilitation research and the factors influencing it. Two studies even include private hospitals [18,19], but much of it is based on other countries [20,21], which are difficult to compare with UK services. Research carried out in the UK tends to prioritise early recovery and is entirely NHS-based, causing some potential problems:

(i) The evaluation of UK hip fracture treatment is based on incomplete data. In 2015, only 57.3% patients with hip fractures had a rehabilitation record [15]. Patient characteristics, including socioeconomic status, are associated with an increased risk of hip fracture and might impact access to private rehabilitation [20]. Patients who utilise private rehabilitation are recorded as being discharged into care, disregarding whether they later return home [15]. These data are used by NHS commissioning groups to determine the clinical/cost effectiveness of treatment, while the National Hip Fracture Database also perform audits for service development recommendations. Incomplete data may camouflage inefficiency, resulting in poor quality treatment and impaired policy/management decisions.
(ii) Anecdotal evidence indicates that private rehabilitation processes are obscured, private facilities may fail to appreciate the services required and new clients remain ignorant of likely progress and costings, while staff and family struggle to set realistic goals and/or meet discharge needs.
(iii) The understanding of patient perspectives remains limited [16]. Anecdotal evidence shows that patients with hip fractures often expect a similar recovery experience to patients with hip replacements and are disappointed when theirs is longer and less restorative. Private patients’ chosen lengths of stay may reflect desirable treatment times; possibly, NHS rehabilitation is too short for full treatment benefit, causing greater long-term healthcare use [18].

The present study examined factors affecting the length of stay (LoS) and discharge destination (DD) of patients with hip fractures in a private rehabilitation setting. The purpose was to increase the evidence base of their treatment and facilitate discharge planning and discuss the possible impact of private rehabilitation data on NHS figures. LoS is a common outcome measure within healthcare research. For the UK Department of Health, it represents local health service efficiency [1,22], with definable conditions, such as hip fractures, used as LoS markers for inter-hospital comparisons [1]. Recent awareness that reducing LoS is not always better for quality of care and long-term cost effectiveness [23] has deprioritised it in national policies but, within rehabilitation, optimal LoS remains a primary focus. This study used a single site to test the suitability of the independent variables, measurement options and data collection methods, whilst reducing the effect of extraneous variables related to individual facility characteristics, such as care culture [24].

## 2. Methods

This was a correlational study incorporating archival data, collected retrospectively from the medical, nursing and physiotherapy records of 56 patients with hip fracture. The study setting was a private elderly care facility (Hospital X) in Oxford, UK. At the time, it had 40 single bedrooms, with approximately 12 for short-stay residents receiving respite, rehabilitation or palliative care. Unusually for a care setting, it had an in-house physiotherapy department.

### 2.1. Study Sample

The records of patients with hip fracture admitted between January 2012 and March 2019 were identified using the physiotherapy department’s admissions files. Data were collected from 56 patient records; all records of patients with hip fractures from this period were eligible.

Ethical approval was obtained from the University of Southampton. Access to the records was granted by the Hospital’s Data Officer.

### 2.2. Variables

#### Study Outcomes

Length of stay (LoS) was measured in calendar days. The LoS of long-stay patients was set at 120 days to reflect recommended follow-up time by NHS Trusts, believing this to be the usual recovery time and the point at which most patients may have moved on to their long-term place of residence [15]. Patients who died within this time, whilst resident, were recorded as long-term care, but with LoS equal to residency.

Discharge destination (DD) was categorised as either home or long-term care.

### 2.3. Independent Variables

To address the study objectives, data on 12 demographic, medical and physical variables were collected from each set of records. These variables were from a selection thought to be potentially influential to LoS and/or DD, informed by a literature review and a patient and public involvement (PPI) group discussion. Quantity was thought to be more relevant than focusing on a few key variables. Variables were selected on the basis of data availability and attempts were made to represent each of the 4 variable types thought to be important for LoS prediction tools [22]. The following data were recorded: age, gender, number of chronic comorbidities, delirium, type of fracture treatment, days between fracture and surgery (latency), days between fracture and admission to hospital (acute LoS), a Functional Independence Measure pre-fracture (FIM_pre-fracture_) and on admission (FIM_admission_), home support, stairs at home and number of physiotherapy sessions per week. The Functional Independence Measure (FIM) is a physical ability measure, found to be valid and reliable in many conditions and settings, including patients with hip fracture in inpatient rehabilitation [25,26,27]. In the present study, only the motor component was used, as cognitive data were unavailable.

### 2.4. Bias and Reliability

Various methods were employed to reduce bias and confirm reliability. The researcher could not be blinded to outcome so, to mitigate this, LoS and DD were the last items collected from the archival data. Confounders were inevitable, particularly as data were collected from a 6.5-year period and surgery/acute care was at a range of hospitals. Their influence was partially offset by including several variables.

The Functional Independence Measure (FIM) was scored retrospectively, as it was not currently included in the initial assessment at the hospital. Self-reporting is thought to be valid for populations without cognitive/communication deficits [28], particularly for motor score, but retrospective scoring appears to be previously unvalidated. Therefore, inter-rater and test–retest reliability tests using intraclass correlation coefficients (ICC) were carried out, each using 6 patient records. As FIM’s 13 variables were individually correlated, a minimum sample size of 3 was required [29]. The records were chosen randomly, taking the first 6 from an Excel Rand()-shuffled dataset. Inter-rater agreement was assessed between FIM scores of the researcher and 2 physiotherapy colleagues; the test–retest scoring was performed by the researcher at a 3-month interval. An ICC > 0.8 indicating a very good agreement level [30] was required, and this was chosen to reflect assumed ease of retrospective FIM scoring.

### 2.5. Statistical Methods

SPSS Version 25.0 (IBM, Armonk, NY, USA) was used for data analysis. Histograms and Shapiro–Wilk tests showed that data were not normally distributed and, hence, median, ranges and non-parametric tests were utilised. Relationships were examined using Spearman’s rho, Mann–Whitney U, Kruskal–Wallis H and chi-squared tests, as appropriate for the data types. Using Mann–Whitney U to test a categorical outcome variable (DD) against a continuous predictor variable (e.g., age) reverses the norm but effectively showed predictor differences across the 2 DD groups.

Further detail was gained using odds ratios, distribution quartiles and Kaplan–Meier survivor analysis. Kaplan–Meier curves indicated the probability of not returning home, depending on the predictor variable. Variables were divided using descriptive categories (FIM scores/fracture treatment) or equal-width groups. Differences between curves, due to being non-parallel and/or crossing, were tested using a Tyrone–Ware test and, if significant (accepted at *p* = 0.05 ÷ number of factors), underwent pairwise log-rank comparisons.

## 3. Results

### 3.1. Sample Characteristics

Table 1 outlines the sample characteristics, including median and ranges. Of particular relevance was that 82% returned home, following an average LoS of 20.5 days. Of the 28% of men and 14% of women who did not return home, 33% (6.5% of the total) died within the first 2 months and the rest remained as long-term residents.

Excellent agreement levels (ICC > 0.9) and small confidence intervals were achieved between FIM scores in all cases except one, ICC = 0.726 (95% CI, 0.481–0.866), when an electronic records system failure meant less information was available for the second test–retest scoring.

### 3.2. Factors Affecting Length of Stay (LoS)

Significant, positive and moderate correlations existed between LoS and age (r_s_(61) = 0.365, *p* = 0.004) and comorbidities (r_s_(61) = 0.332, *p* = 0.009), whilst LoS had significant negative relationships with FIM_admission_ (r_s_(61) = −0.414, *p* = 0.001) and FIM_%change_ (r_s_(61) = −0.299, *p* = 0.019). Surprisingly, no correlation was found with acute LoS (r_s_ (61) = 0.220, *p* = 0.088) nor support at home (*p* = 0.440) (Table 2).

### 3.3. Factors Affecting Discharge Destination (DD)

Several variables were significantly associated with returning home. Generally, participants were younger (mean rank home = 27.99, long-stay = 43.29; U = 146.500, z = −2.680, *p* = 0.007), were less likely to have delirium (OR 0.195 (CI 95%, 0.047 to 0.817)) and had fewer co-morbidities (mean ranks 27.39 and 45.75; U = 117.000, z = 3.276, *p* = 0.001). FIM_pre-fracture_ and FIM_admission_ were both higher (U = 95.000, *p* = 0.000 (identical scores)), differences which were clearly evident in the group distribution. Whilst both FIM_pre-fracture_ groups had a similar range, the distribution was skewed in opposite directions, with percentiles partially overlapping at 75%/25%. FIM_admission_ percentiles overlapped similarly, but the ranges also only partially overlapped.

### 3.4. Survivor Analysis

Significant differences were found in the survival distributions relating to age (c^2^ = 16.493, *p* = 0.001)**,** specifically for the 60–69 years and 90–99 years groups (c^2^ = 13.105, *p* = 0.000) and FIM_admission_ (c^2^ = 14.786, *p* = 0.005), specifically for the 27–38 vs. 39–62 groups (c^2^ = 6.721, *p* = 0.010) and 27–38 vs. 63–77 groups (c^2^ = 7.856, *p* = 0.005). The first and last FIM_admission_ groups were too small for comparison.

**Figure 1:**
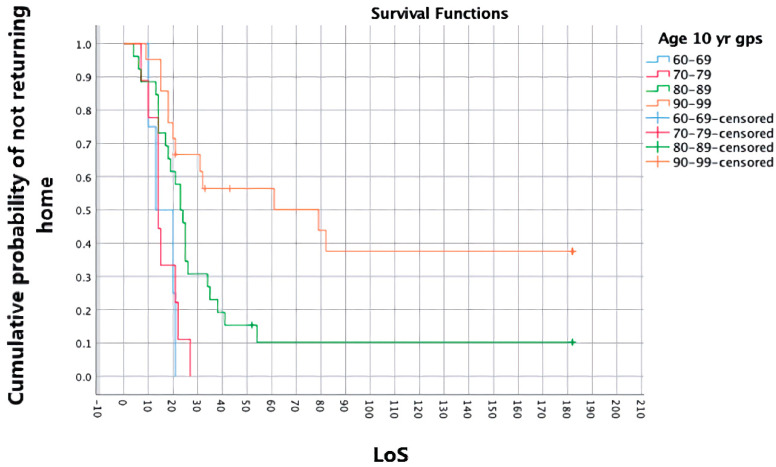
Survivor analysis curve—age (LoS in days).

**Figure 2:**
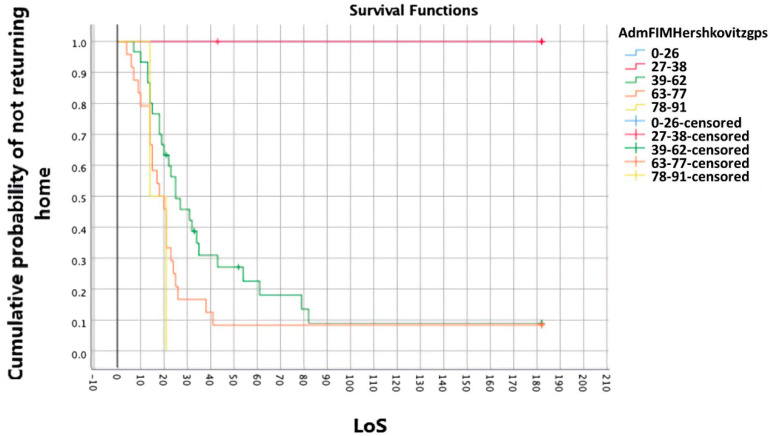
Survivor analysis curve—FIM_admission_ (LoS in days).

Clinical interpretations of the Kaplan–Meier curves can be gained from chart depictions—see Figure 1 and Figure 2 for the survival distributions of age and FIM_admission_. For example, 50% of the sample went home before 21 days, including all patients aged < 69 or with an FIM_admission_ score > 78. A patient aged < 80 and/or with an FIM_admission_ of 63–78 was unlikely to stay longer than 28 days. However, a patient aged 90–99 had a 57% risk of not going home within 60 days. All home discharges were completed within 90 days.

## 4. Discussion

The present study utilised a private, rather than public, facility to examine the length of stay (LoS) and discharge destination (DD) of hip fracture rehabilitation patients in the UK. It particularly builds on research which notes that either one or both of these outcomes are affected by FIM_admission_ [31,32,33,34] and supports studies which identify age [31,32,35,36,37], co-morbidities [38] and latency [32,36] as influential. Other influential factors noted were delirium and FIM_pre-fracture_, previously only reported in studies of acute care [39,40]. No variable had strong correlations with the outcomes, supporting the suggestion that factors have a compound effect [37]. Evidence could not be found of suggestions that gender, fracture treatment and support are important [32,34,37,38].

Similar hip fracture studies included sample sizes ranging from 54 to 117,168 and utilised retrospective or prospective data collection to investigate LoS [19,37,41], DD [32,35,36,38] or both [31,34]. Age and gender distributions were relatively congruent, suggesting that a typical patient with a hip fracture—female and octogenarian—is the same worldwide. However, an average LoS range of 7.0–34.4 days from seven studies [19,31,32,34,37,41] and a discharged home range of 60.4–88.0% from six studies [31,32,35,36,38,41] may reflect the range of cognitive and physical abilities represented in these studies, as well as different care cultures. The current study sample, despite having the highest average age, was in the upper range for both outcomes. It possibly benefitted from comparatively high FIM_admission_ and its self-funding status gave it motivational [42] and socioeconomic advantages [43], although a private/public comparison is made difficult by a lack of detail regarding the study setting in other studies. The use of means by most of these studies should also be recognised, as they are larger than the median on typically positively skewed LoS histograms. Calculated for comparison, the present study’s mean was excessively high, owing to its long-stay subgroup for LoS. This suggests that, in the other studies, discharge to long-term care occurred sooner than the nominal 4 months found in this study, possibly indicating insufficient recovery time, thereby explaining lower discharge home figures.

Compared to NHS mean averages [4], this study’s population were less likely to have delirium and more likely to be discharged home but had longer acute and super-spell stays. Cameron et al. [19] noted that rehabilitation LoS was longer in ‘off-site’ facilities than in acute hospitals and related this to less-efficient care cultures. The differences in this comparison may be linked with population demographics, socioeconomic status and affordability, and the likelihood that private rehabilitation attracts those in the middle of the health/ability scale, rather than the whole range treated by the NHS. An element of patient choice related to discharge is also thought to be likely. However, by choosing private rehabilitation, this study’s population, for the purposes of NHS data, has a final residency of nursing home placement [15]. If these differences occur throughout private care, a potential to change NHS results exists for total treatment time, residence at 120 days (a NHFD key performance indicator) and 30-day mortality. Physiotherapists have proved themselves willing to facilitate research data collection [44], and asking private facilities to submit 120-day data might improve data accuracy.

For gauging approximate LoS and probable DD, FIM_admission_ is likely the most useful variable tested in this study. In accordance with similar research [22,31,34,38,41], it influenced both LoS and DD and had the highest correlation with each. If restricted on time, its self-care section may be sufficient [18,45]. Survivor analysis provides one way of using FIM_admission_ or any of the predictor variables for estimating LoS/DD. Combining the survival pattern of a variable subgroup, e.g., FIM_admission_ scores of 78–91, with the average LoS of that subgroup would provide the most personalised recovery estimate possible from this study’s data, though it leaves much room for error. The categorisation method, human idiosyncrasies and personal circumstances are likely to act as confounders, which must be considered. FIM_admission_ may have additional functional uses: categorisation according to dependency level can identify the most suitable treatment approach for discharge within a fixed time [31]. Tan and Saw [41] recommend a weekly rehabilitation goal of 7+ FIM (motor) points, encouraging comprehensive treatment plans, although a smaller goal might better suit this study’s older sample, e.g., the 0.4 FIM (motor) points achieved/day by Hershkovitz et al. [34].

Recognising that patients recover at different speeds may explain why discharge home failed to correlate with a shorter LoS. Older and less healthy participants generally had a longer LoS, suggesting slower recoveries, but not necessarily less successful recoveries. Improvement can continue for many months [18,46], indicating that these patients might benefit from community services referral on discharge. Patients with delirium need not have limited recovery either. In this study, they were five times more likely to need nursing home placement, which is comparable to 2017 UK figures [4], and were eight times more likely to die within a year. However, with prompt screening, staff training and individualised care, patients with hip fractures of all cognitive levels might achieve the same FIM gains in the same LoS [47]. Symptoms must be resolved within 1 month for full recovery [48]. FIM and delirium screening, e.g., mini-mental state examination [49] or 4AT [50,51,52], could form part of an initial/ongoing assessment.

Archival data collection from patient records occasionally revealed recording errors, including incorrect personal details, dates, and outcomes. Other errors may have gone unnoticed. Predictor variables relating to hospital characteristics, socio-economic factors, and health-related quality of life are recommended [22,52].

Sample size was a limitation because it was insufficient for regression analysis and increased prediction ability. The potential impact of those who died may need to be considered. Extending the study to other private rehabilitation facilities would be necessary to allow generalisations to be made. This would require extensive coordination and the cooperation of many, usually independent, facilities. Inter-facility comparison could be facilitated by standardising predictor variables, e.g., 4AT test for delirium and the Geriatric Cumulative Illness Rating Scale (CIRS-G) [53] for comorbidities, and introducing discharge measures, e.g., FIM_discharge_.

## 5. Conclusions

The present study examined factors affecting the length of stay (LoS) and discharge destination (DD) of patients recovering from hip fracture surgery at a private UK rehabilitation facility. The results confirmed that patients with hip fractures were heterogeneous, recovering at different speeds and with different results. There are factors available on admission which can strongly indicate a patient’s potential LoS and DD, but no factor was indicative on its own. Regression analysis is required for greater precision in prediction. However, results suggested that discharge destination will be evident by 90 days, providing patients and their families with a measure by which to plan long-term residency. Recommendations for immediate implementation into practice include the measurement on admission of delirium and functional ability. These might facilitate specific goal setting more promptly than the current trend, for both care and therapy staff.

## Figures and Tables

**Table 1:** Characteristics of study sample.

Variable	All	Home (DD = 1)	Long-Term Care (DD = 2)
Age, median (IQR)	87.0	86.0 (8)	92.5 (5.3)
Gender, *n* = male (%)	18 (29.5)	12 (66.7)	6 (50)
Comorbidities, median (IQR)	4.0	3.0 (2.25)	5.5 (3)
Delirium, *n* = Yes (%)	11 (18)	6	5
Fracture treatment, *n* (%)			
Fixation	12 (19.7)	10 (20.4)	2 (16.7)
Mobile fixation	20 (32.8)	16 (32.7)	4 (33.3)
Arthroplasty	27 (44.3)	22 (44.9)	5 (41.7)
Conservative	2 (3.3)	1 (2.0)	1 (8.3)
Latency, median (IQR) (days fracture to treatment)	1.0 (1.0)	1.0 (1.0)	2.0 (7.0)
LoS_acute_, median (IQR)	15.0 (10.5)	15.0 (9.5)	19.5 (25.5)
FIM_pre-fracture_, median (IQR)	88.0 (7.0)	89.0 (5.0)	81.0 (22.0)
FIM_admission_, median (IQR)	61.0 (24.0)	62.0 (9.5)	46.0 (21.75)
FIM_%change_, median (IQR)	29.7 (11.7)	29.7 (11.1)	32.0 (30.1)
Home support, *n* (%)			
Live-in	23 (37.7)	18 (36.7)	5 (41.7)
Visiting	28 (45.9)	23 (46.9)	5 (41.7)
Alone	9 (14.8)	8 (16.3)	1 (8.3)
Dependent spouse	1 (1.6)	0 (0.0)	1 (8.3)
Stairs, *n* = Yes (%)	36 (59.0)	29 (59.2)	7 (58.3)
Physio sessions/wk, median (IQR)	5 (2.0)	5 (2.0)	5 (1.75)
Length of stay (LoS), median (IQR)	22.0 (27.5)	20.0 (12.5)	182.0 (136.75)

**Table 2:** Factors associated with length of stay and discharge destination.

Independent Variable	Tests	Outcome	
		LoS	DD
Age	Spearman’s r/Mann-W U	cc 0.365, *p* = 0.004 **	U = 146.500, *p* = 0.007 **
Gender	Mann–W U/Chi^2^ (2 × 2)	U = 298.00, *p* = 0.158	c^2^ = 3.016, *p* = 0.082
Co-morbidities	Mann–W U/Chi^2^ (2 × 2)	cc 0.332, *p* = 0.009 **	c^2^ = 11.680, *p* = 0.020 *
Delirium	Mann–W U/Chi^2^ (2 × 2)	U = 197.00, *p* = 0.143	c^2^ = 5.645, *p* = 0.018 *
Fracture treatment	Kruskal–Wallis H/Chi^2^ (r × c)	*p* = 0.251	c^2^ = 1.257, *p* = 0.739
Latency	Spearman’s r/Mann–W U	cc −0.085, *p* = 0.521	U = 208.5, *p* = 0.431
^LoS^acute	Spearman’s r/Mann–W U	cc 0.220, *p* = 0.088	U = 181.500, *p* = 0.041 *
^FIM^pre-fracture	Spearman’s r/Mann–W U	cc 0.242, *p* = 0.062	U = 95.500, *p* = 0.000 **
^FIM^admission	Spearman’s r/Mann–W U	cc 0.414, *p* = 0.001 **	U = 95.500, *p* = 0.000 **
^FIM^%change	Spearman’s r/Mann–W U	cc 0.299, *p* = 0.019 *	U = 217.00, *p* = 0.162
Stairs	Mann–W U/Chi^2^ (2 × 2)	U = 429.00, *p* = 0.758	c^2^ = 0.003, *p* = 0.957
Support	Kruskal–Wallis H/Chi^2^ (r × c)	*p* = 0.440	c^2^ = 4.621, *p* = 0.536
Treatment/wk	Spearman’s r/Mann–W U	cc 0.170, *p* = 0.191	U = 264.500, *p* = 0.839

* Significant at *p* = 0.05 level; ** significant at *p* = 0.01 level.

## Data Availability

The data for this project are confidential but may be obtained through Data Use Agreements with the University of Southampton. Researchers interested in access to the data may contact the author, who will assist with any reasonable replication attempts for two years following publication.

## References

[1] Royal College of Physicians National Hip Fracture Database (NHFD) Annual Report 2017. https://nhfd.co.uk/files/2017ReportFiles/NHFD-AnnualReport2017.pdf.

[2] Royal College of Physicians National Hip Fracture Database National Report 2011. https://www.nhfd.co.uk/20/hipfractureR.nsf/945b5efcb3f9117580257ebb0069c820/53e0ba402226ef27802578c500308c66/$FILE/NHFDNationalReport2011Final.pdf.

[3] Schulz C., Büchele G., Rehm M., Rothenbacher D., Roigk P., Rapp K., Günster C., König H.-H., Reber K. (2019). Patient characteristics as indicator for care dependence after hip fracture: A retrospective cohort study using health insurance claims data from Germany. J. Am. Med. Dir. Assoc..

[4] Royal College of Physicians (2018). National Hip Fracture Database Annual Report 2018.

[5] Handoll H.H.G., Sherrington C., Mak J.C.S. (2011). Interventions for improving mobility after hip fracture surgery in adults. Cochrane Database Syst. Rev..

[6] Royal College of Physicians (2011). Hip Fracture: Management—NICE Guideline. https://www.rcplondon.ac.uk/guidelines-policy/hip-fracture-management-nice-guideline.

[7] Oldmeadow L.B., Edwards E.R., Kimmel L., Kipen E., Robertson V.J., Bailey M. (2006). No rest for the wounded: Early ambulation after hip surgery accelerates recovery. ANJ J. Surg..

[8] Gerety M.B., Soderholm-Difatte V., Winograd C.H. (1989). Impact of prospective payment and discharge location on the outcome of hip fracture. J. Gen. Intern. Med..

[9] Dodds C., Foo I., Jones K., Singh S.K., Waldmann C. (2013). Peri-operative care of elderly patents—An urgent need for change: A consensus statement to provide guidance for specialist and non-specialist anaesthetists. Periopr. Med..

[10] Kondo A., Zierler B.K., Hagino H. (2010). Relationship between the length of hospital stay after hip fracture surgery and ambulatory ability or mortality after discharge in Japan. Jpn. J. Nurs. Sci..

[11] Kronborg L., Bandholm T., Palm H., Kehlet H., Kristensen M.T. (2016). Physical activity in the acute ward following hip fracture surgery is associated with less fear of falling. J. Ageing Phys. Act..

[12] Kristensen M.T. (2011). Factors affecting functional prognosis of patients with hip fracture. Eur. J. Phys. Rehabil. Med..

[13] Royal College of Physicians National Hip Fracture Database Annual Report 2016. RCP: London, UK.

[14] Chartered Society of Physiotherapy (2018). Hip Fracture Rehabilitation in Physiotherapy Practice: From Hospital to Home. https://www.csp.org.uk/system/files/publication_files/001563_Hip%20Fracture%20Standards_Full%20version_A4_V4_5-8-19_0.pdf.

[15] Wakeman R. (2017). Improving Our Understanding of the Care and Rehabilitation of Hip Fracture Patients.

[16] Griffiths F., Mason V., Boardman F., Dennick K., Haywood K., Achten J., Parsons N., Griifin X., Costa M. (2014). Evaluaing recovery following hip fracture: A qualitative interview study of what is important to patients. BMJ Open.

[17] GK Strategy (2017). A Private Choice: The Changing Face of the UK Health Market. https://gkstrategy.com/report/uk-health-a-private-choice/.

[18] Mallinson T., Deutsch A., Bateman J., Tseng H.-Y., Manheim L., Almagor O., Heinemann A.W. (2014). Comparison of discharge functional status after rehabilitation in skilled nursing, home health and medical rehabilitation facilities for patients after hip fracture. Arch. Phys. Med. Rehabil..

[19] Cameron L.D., Kurrle S., March L. (1998). Rehabilitation length of stay after hip fracture. Aust. N. Z. J. Med..

[20] Richards T., Glendenning A., Benson D., Alexander S., Thati S. (2018). The independent patient factors that affect length of stay following hip fractures. Ann. R. Coll. Surg. Engl..

[21] Su B., Newson R., Soljak H., Soljak M. (2018). Associations between post-operative rehabilitation of hip fracture and outcomes: National database analysis. BMC Musculoskelet. Disord..

[22] Lu M., Sajobi T., Lucyk K., Lorenzetti D., Quan H. (2015). Systematic review of risk adjustment models of hospital length of stay (LOS). Med. Care.

[23] Clarke A. (2002). Length of in-hospital stay and its relationship to quality of care. BMJ Qual. Saf..

[24] Lindberg L., Ekstrom W., Hedstrom M., Flodin L., Lofgren S., Ryd L. (2017). Changing caring behaviours in rehabilitation after a hip fracture—A tool for empowerment?. Psychol. Health Med..

[25] Dodds T.A., Martin D.P., Stolov W.C., Deyo R.A. (1993). A validation of the functional independence measurement and its performance among rehabilitation inpatients. Arch. Phys. Med. Rehabil..

[26] Petrella R.J., Overend T., Chesworth B. (2002). FIM after hip fracture. Am. J. Phys. Med. Rehabil..

[27] Gerrard P., Goldstein R., DiVita M.A., Slocum C., Ryan C.M., Mix J., Niewczyk P., Kazis L., Zafonte R., Schneider J.C. (2015). Functional status and readmissions in unilateral hip fractures. Am. J. Manag. Care.

[28] Vadassery S.J., Kong K.H., Ho W.M.L., Seneviratna A. (2019). Interview functional independence measure score: Self-reporting as a simpler alternative to multidisciplinary functional assessment. Singap. Med. J..

[29] Bujang M.A., Baharum N. (2017). A simplified guide to determination of sample size requirement for estimating the value of intraclass correlation coefficient: A review. Arch. Orofac. Sci..

[30] Koo T.K., Li M.Y. (2016). A guideline of selecting and reporting intraclass correlation coefficients for reliability research. J. Chiropr Med..

[31] Chin R.P.H., Ng B.H.P., Cheung L.R.C. (2008). Factors predicting rehabilitation outcomes of elderly patients with hip fractures. Hong Kong Med. J..

[32] Canadian Institute for Health Information (2015). Factors Predicting Return Home from Inpatient Rehabilitation Following Hip Fracture Surgery. https://secure.cihi.ca/free_products?NRS_Hips_2015_EN_web.pdf.

[33] Ariza-Vega P., Jimenez-Moleon J.J., Kristensen M.T. (2012). Change of residence and functional status within three months and one year following hip fracture surgery. Disabil. Rehabil..

[34] Hershkovitz A., Kaladariov Z., Hermush V., Weiss R., Brill S. (2007). Factors affecting short-term rehabilitation outcomes of disabled elderly patients with proximal hip fracture. Arch. Phys. Med. Rehabil..

[35] Hayashi H., Iwai M., Matsuoka H., Nakashima D., Nakamura S., Kubo A., Tomiyama N. (2016). Factors affecting the discharge destination of hip fracture patient who live alone and have been admitted to an inpatient rehabilitation unit. J. Phys. Ther. Sci..

[36] Cree A.K., Nade S. (1999). How to predict return to the community after fracture of proximal femur in the elderly. Aust. N. Z. J. Surg..

[37] Ireland A.W., Kelly P.J., Cumming R.G. (2015). Total hospital stay for hip fracture measuring the variations due to pre-fracture residence, rehabilitation, complications and comorbidities. BMC Health Serv. Res..

[38] Wang C.Y., Graham J.E., Karmarkar A.M., Reistetter T.A., Protas E.J., Ottenbacher K.J. (2014). FIM motor scores for classifying community discharge after inpatient rehabilitation for hip fracture. PM&R.

[39] Edelstein D.M., Aharanoff G.B., Karp A., Capla E.L., Zuckerman J.D., Koval K.J. (2004). Effect of postoperative delirium on outcome after hip fracture. Clin. Orthop. Relat. Res..

[40] Everink I.H.J., van Haastregt J.C.M., van Hoof S.J.M., Schols J.M.G.A., Kempen G.I.J.M. (2016). Factors influencing home discharge after inpatient rehabilitation of older patients: A systematic review. BMC Geriatr..

[41] Tan Y.-L., Saw H.-M. (2016). Hip fractures: A review of predictors affecting FIM, ambulation & rehabilitation length of stay during inpatient rehabilitation at Singapore General Hospital. Proc. Singap. Healthc..

[42] Kolt G.S., McEvoy J.F. (2003). Adherence to rehabilitation in patients with low back pain. Man. Ther..

[43] Quah C., Boulton C., Moran C. (2011). The influence of socioeconomic status on the incidence, outcome and mortality of fractures of the hip. J. Bone Joint Surg..

[44] Royal College of Physicians (2018). Recovering After a Hip Fracture: Helping People Understand Physiotherapy in the NHS.

[45] Amundson J., Brunner A., Huffman S. (2004). FIM scores as an indicator of length of stay and discharge destimation in CVA patients: A retroactive outcomes study. https://www.semanticscholar.org/paper/FIM-Scores-as-an-Indicator-of-Length-of-Stay-and-in-Amundson-Brunner/3e8048bc33e1dd94e4beeacd56b7a08ae3072650.

[46] Auais M.A., Eilayyan O., Mayo N.E. (2012). Extended exercise rehabilitation after hip fracture improves patients’ physical function: A systematic review and meta-analysis. Phys. Ther..

[47] McGilton K.S., Mahomed N., Davis A.M., Flannery J., Calabrese S. (2009). Outcomes for older adults in an inpatient rehabilitation facility following hip fracture surgery. Arch. Gerontol. Geriatr..

[48] Kiely D.K., Bergmann M.A., Jones R.N., Murphy K.M., Orav E.J., Marcantonio E.R. (2004). Characteristics associated with delirium persistence among newly admitted post-acute facility patients. J. Gerentol. Biol. Sci. Med. Sci..

[49] Ringdal G.I., Ringdal K., Juliebo V., Wyller T.B., Hjermstad M.J., Loge J.H. (2011). Using the mini- mental state examination to screen for delirium in elderly patients with hip fracture. Dement. Geriatr. Cogn. Disord..

[50] Bellilli G., Morandi A., Davis D.H.J., Mazzola P., Turco R., Gentile S., Ryan T., Cash H., Guerini F., Torpilliesi T. (2014). Validation of the 4AT, a new instrument for rapid delirium screening: A study in 234 hospitalised older people. Age Ageing.

[51] MacLullich A.M.J. (2011). 4AT Rapid Clinical Test for Delirium. https://www.the4at.com/authors.

[52] Harrell M.C., Bradley M.A. (2009). Data Collection Methods: Semi-Structured Interviews and Focus Groups.

[53] Sajobi T., Lu M., Jiang J., Quan H. (2017). Improving the accuracy of length of stay risk adjustment models using linked data. Int. J. Popul. Data Sci..

